# Distinct Responses to Pathogenic and Symbionic Microorganisms: The Role of Plant Immunity

**DOI:** 10.3390/ijms231810427

**Published:** 2022-09-09

**Authors:** Li Ji, Xiangrui Yang, Feifei Qi

**Affiliations:** Shandong Provincial Key Laboratory of Animal Resistance Biology, Institute of Biomedical Sciences, College of Life Sciences, Shandong Normal University, Jinan 250014, China

**Keywords:** hormone, innate immunity, plant–microbe interaction, symbiosis, sRNA

## Abstract

Plants must balance both beneficial (symbiotic) and pathogenic challenges from microorganisms, the former benefitting the plant and agriculture and the latter causing disease and economic harm. Plant innate immunity describes a highly conserved set of defense mechanisms that play pivotal roles in sensing immunogenic signals associated with both symbiotic and pathogenic microbes and subsequent downstream activation of signaling effector networks that protect the plant. An intriguing question is how the innate immune system distinguishes “friends” from “foes”. Here, we summarize recent advances in our understanding of the role and spectrum of innate immunity in recognizing and responding to different microbes. In addition, we also review some of the strategies used by microbes to manipulate plant signaling pathways and thus evade immunity, with emphasis on the use of effector proteins and micro-RNAs (miRNAs). Furthermore, we discuss potential questions that need addressing to advance the field of plant–microbe interactions.

## 1. Introduction

The constant exposure of plants to microorganisms in the surrounding environment can result in disease and consequent severe economic damage to agriculture [1]. To protect themselves from pathogen attacks, plants have evolved a sophisticated, multilayered immune system involving constitutive and inducible defenses to detect pathogenic microbes [2]. Unlike vertebrates, plants mainly rely on innate immunity to combat pathogens due to their lack of an adaptive immune system.

Plants detect microbes through recognition of specific microbial structures by pattern recognition receptors (PRRs), which always localize at the plasma membrane and work with coreceptors [3]. PRRs consist of receptor-like kinases (RLKs) and receptor-like proteins (RLPs): RLKs possess an intracellular kinase domain, a transmembrane domain, and an extracellular domain, while RLPs lack the intracellular domain but contain the other two domains [4,5,6,7]. RLKs and RLPs, as the first defense barrier, detect microbe-associated molecular patterns (MAMPs) to initiate pattern-triggered immunity (PTI). However, some pathogens can evade PTI by secreting effector proteins into plant cells to interfere with MAMP detection and/or subsequent signal transduction, with consequent disease development. Plants have in turn evolved another robust and rapid immune response, namely effector-triggered immunity (ETI), to detect pathogen effectors, a process that depends on intracellular nucleotide-binding leucine-rich repeat (NLR) proteins [8]. Plant NLRs comprise a C-terminal leucine-rich repeat (LRR) domain, a central nucleotide-binding (NB-ARC) domain, and an N-terminal domain that may be a coiled-coil domain, a resistance to powdery mildew 8 (RPW8) domain, or a toll/interleukin-1 receptor homology (TIR) domain [9,10]. Recently, the structures of some NLRs have been resolved, providing a basis for understanding plant NLRs [9,11,12,13]. Generally, PRRs function as extracellular immune receptors while NLRs mediate intracellular defenses. Together, PRRs and NLRs cooperate to defend against invaders.

However, some microorganisms can establish a symbiotic relationship with the plant that is beneficial to the plant host. For example, arbuscular mycorrhizal (AM) fungi collaborate with most land plants to improve their phosphorus nutrition through the establishment of symbiotic interactions. In addition, another widely studied symbiosis is the relationship between rhizobia and legume plants, in which rhizobia fix atmospheric nitrogen and transfer it to the plant. In these cases, beneficial microbes are subjected to the plant immune system as pathogens, but they could survive. However, the underlying mechanism(s) dictating this outcome remains uncertain. Is symbiosis a result of an absent immune response upon recognition of beneficial microbes or do symbiotic microbes overcome plant defense mechanisms? How plants distinguish “friend” from “foe” is a further intriguing question, particularly as MAMPs are conserved in both symbiotic and pathogenic microbes [14]. Emerging evidence suggests that the establishment of symbiotic interactions is an intricate process that requires contributions from both plants and microorganisms. In this review, we highlight the importance of plant immunity in the recognition of different microbes and discuss how plant immune pathways are tightly manipulated in symbiotic relationships, focusing on the association between plants and AM fungi and rhizobia. Understanding the molecular mechanisms underpinning immune responses and tolerance will give us some guidance to foster beneficial microbial symbiosis while repudiating pathogenic interaction and thus, ultimately, contribute to improving crop production.

## 2. The Plant Immune System

The plant immune system includes local immunity with PTI and ETI as well as systemic immunity with systemic acquired resistance (SAR) and induced systemic immunity (ISR). Systemic immunity refers to systemic signals spread from the affected site to unaffected tissues to enhance resistance to a subsequent secondary stress. For example, the differentiated outer cell layers display low protein level of PRRs and deficient PTI in *Arabidopsis thaliana*. However, these cells could be induced to highly express PRRs and be responsive to pathogens by neighbor cell damage, suggesting that a local immune response could be induced in nonresponsive cells [15]. SAR is triggered by pathogens and is primarily associated with salicylic acid (SA)-dependent signaling [16], whereas ISR is induced by beneficial microbes with jasmonic acid (JA) or ethylene (ET) signaling independent of SA [17]. The SA and JA pathways have a well-documented antagonistic relationship [18]. In addition, a novel immune strategy, termed general non-self response (GNSR), has been identified in *Arabidopsis thaliana*. GNSR involves a core set of 24 genes that are consistently induced in plant responses to the microbiome, indicating that plants use their immune systems to shape their microbiota [19]. Here, we focus on how the plant innate immunity functions appropriately during its interactions with microbes.

**Figure 1:**
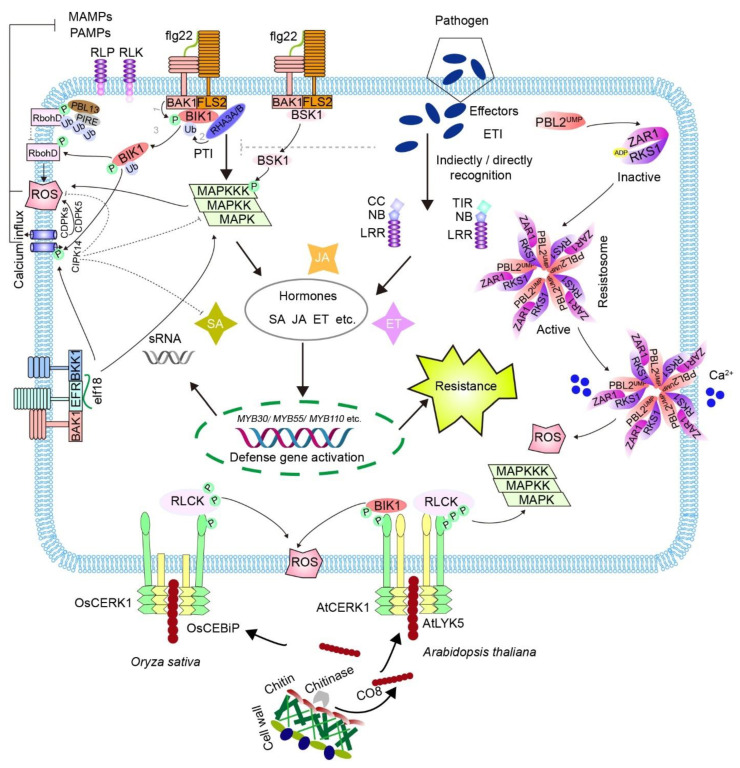
Schematic summary of plant innate immune signaling pathways. MAMPs, sensed by membrane-localized PRRs which work with RLCKs, trigger immune responses, which typically involve MAPK cascades, a ROS burst, a Ca^2+^ influx, and upregulation of transcription factors such as MYB30, MYB55, and MYB110 to induce defense gene expression. For example, upon flg22 detection, BIK1 is phosphorylated by FLS2-BAK1, followed by monoubiquitination by RHA3A/B and then dissolution from the FLS2-BAK1 complex to phosphorylates RbohD to promote ROS production and cyclic nucleotide-gated channels to increase the concentration of cyclic calcium. ROS production is negatively regulated by the PBL13-PIRE complex which phosphorylates and ubiquitinates RbohD. Additionally, BSK1 disassociates from the FLS2-BAK1 complex when flg22 is detected and subsequently phosphorylates MAPKKK to induce downstream signals. In addition, Ca^2+^-dependent kinases (CDPKs), such as CDPK5, have been shown to be involved in PTI by activating NADPH oxidases. However, another Ca^2+^ sensor, CBL-interacting protein kinase 14 (CIPK14), negatively regulates MAPK-mediated immune responses and SA production. Furthermore, the EFR/BAK1/BKK1 complex shares common signaling pathways with FLS2 in response to elf18. In addition, fungal chitin is hydrolyzed by chitinase and releases COs to trigger an immune response. For example, CO8 is recognized by AtCERK/AtLYK5 and OsCERK/OsCEBiP in *Arabidopsis thaliana* and *Oryza sativa*, respectively. AtCERK/AtLYK5 and OsCERK/OsCEBiP associates with their RLCKs to initiate downstream immune responses, including a ROS burst and activation of an MPK cascade. Some pathogens secrete effector proteins into the host cell to interfere with MAMP detection and/or subsequent signal transduction. In response, plants have evolved ETI to detect pathogen effectors, which is mediated by intracellular NLR receptor proteins such as CC-NLR and TIR-NLR. For example, uridylylated PBL2 (PBL2^UMP^) is recruited by the ZAR1-RKS1 complex, which forms the ZAR1-RKS1-PBL2^UMP^ resistosome. The resistosome localizes to the membrane and is controlled by Ca^2+^ to initiate ETI by triggering a ROS burst. Endogenous phytohormones, such as SA, JA, and ET, as well as small RNAs (sRNAs) are also induced and contribute to plant immunity.

Plants detect microbes through MAMPs or microbial effectors. Plenty of MAMPs have been identified, including β-glucans and the eicosapolyenoic acid (EP) of oomycete, bacterial flagellin peptide flg22, peptidoglycan (PGN), lipopolysaccharide (LPS), EF-Tu peptide elf18, lipopeptides, fungal wall component chitin, and chitosan [20]. The first layer of the immune response begins when PRRs sense MAMPs. This then triggers the activation of downstream plasma membrane-associated receptor-like cytoplasmic kinases (RLCKs), followed by a ROS burst, calcium influx, and mitogen-activated protein kinase (MAPK) cascades encompassing a MAPK kinase kinase (MAPKKK), a MAPK kinase (MAPKK), and a MAPK (MPK) (Figure 1) [21,22]. In addition, MPK cascade activation by chitin elicitor induces the expression of MYB transcription factors MYB30, MYB55, and MYB110, resulting in the accumulation of ferulic acid together with enhanced resistance to pathogens in rice [23]. These studies indicate that robust defense response involves multiple alterations in gene expression, protein modifications, and synthesis of secondary metabolites.

Several RLCKs are thought to link PRRs to key downstream signaling pathways, such as RLCK subfamily II components in *Arabidopsis*: botrytis-induced kinase 1 (BIK1), PBS1-like 1 (PBL1), and brassinosteroid signaling kinase 1 (BSK1) [22,24,25,26,27]. The extensively studied bacterial PAMPs that elicit immune responses are flg22 and elf18. For instance, once flg22 is detected, FLS2 instantaneously forms a complex with brassinosteroid insensitive 1-associated kinase 1 (BAK1) and concomitantly interacts with BIK1 [24,25]. Subsequently, BIK1 is phosphorylated by BAK1, followed by monoubiquitination by ring-H2 finger A3A (RHA3A) and RHA3B and then dissolution from the FLS2-BAK1 complex to activate immune signaling [28]. As a serine/threonine kinase, activated BIK1 subsequently phosphorylates the N terminus of NADPH oxidase RbohD to promote ROS production [29,30] and cyclic nucleotide-gated channels to increase the concentration of cyclic calcium [31]. Many calcium-dependent kinases (CDPKs), including CDPK5, are also involved in ROS production [32]. However, another Ca^2+^ sensor, CBL-interacting protein kinase 14 (CIPK14), negatively regulates MAPK-mediated immune responses and SA production [33]. In addition, flg22 activates MAPK cascades to upregulate defense genes [34,35,36]. It has been shown that BSK1, another RLCK member, may transduce upstream cues to activate MAPK cascades. Similar to BIK1, BSK1 associates with FLS2 in the absence of flg22, but disassociates from the complex when flg22 is detected [27]. Activated BSK1 then phosphorylates MAPKKK5 which associates with multiple MAPK kinases to induce downstream signals [36]. Similarly, EFR forms a complex with BAK1 and BAK1-LIKE 1 (BKK1) [37,38] and shares common signaling pathways with FLS2 in response to elf18 [39,40].

Additionally, plants also have powerful mechanisms to sense fungi, which mainly depend on the recognition of chitin. Chitin, a conserved structural component of fungal cell walls, is a polymer of β-1,4-linked N-acetylglucosamine (GlcNAc). To avoid fungal infection, plants secrete chitinases that hydrolyze chitin and release chitooligosaccharides (COs). Chitin hexamers (CO-6) or octamers (CO-8), as breakdown products, are recognized by lysin motif (LysM)-containing RLPs or RLKs to initiate intracellular immune signaling (Figure 1) [41,42]. In *Arabidopsis thaliana*, chitin is detected by the LysM-receptor kinases AtCERK1 and AtLYK5, in which AtLYK5 has a higher affinity for COs, while AtCERK1 is an indispensable coreceptor with modest affinity [43]. In addition, AtLYK4 was shown to be involved in chitin signaling by associating with AtLYK5 [44], or recognizing COs directly [45]. Notably, AtCERK1, but not AtLYK5 or AtLYK4, has kinase activity, suggesting that AtCERK1 is responsible for initiating immune responses. It has been shown that chitin rapidly induces autophosphorylation of AtCERK1 at multiple residues in the kinase and juxtamembrane domains, which is pivotal for chitin-induced signaling [46]. Activated AtCERK1 thus activates downstream members of RLCK subfamily VII which contains 46 members in *Arabidopsis thaliana*, including PBL1 to PBL43, PBS1, CDG1, and BIK1 [24,26,47,48,49,50]. In brief, chitin initiates the removal of BIK1 from AtCERK1, and BIK1 in turn phosphorylates and activates NADPH oxidase and cyclic nucleotide-gated channel proteins, such as CNGC2 and CNGC4, leading to ROS production [29] and increased cytosolic calcium [31]. In addition, RLCK VII-4 subfamily members, including PBL19, could phosphorylate MAPKKK5 to activate MAPKKK5-MKK4/5-MPK3/6 cascades and MEKK1 to promote MPK4 activation and defense gene expression [34]. Moreover, activated MPK3/6 and MPK4 in turn phosphorylate MAPKKK5 and MEKK1, serving as positive feedback mechanisms [34].

In *Oryza sativa*, chitin detection involves OsCEBiP and OsCERK1. Although OsCEBiP binds COs with higher affinity, it does not have a cytoplasmic domain [51]. Considering that OsCERK1 does not associate with chitin but possesses a kinase domain, it is thought that OsCEBiP interacts with chitin and subjects it to its partner OsCERK1 to induce immune signaling [51,52]. Briefly, a single chitin fragment crosslinks two OsCEBiP molecules to promote OsCEBiP dimerization, after which OsCEBiP associates with OsCERK1 to form an active hetero-oligomeric receptor complex, which has been suggested to act as a “sandwich-like” homodimer [52,53]. In this model, OsCERK1 is activated via autophosphorylation and in turn interacts with and phosphorylates OsRLCK185, a member of the RLCK VII family. Subsequently, OsRLCK185 phosphorylates distinct substrates to initiate an immune response [54,55,56,57,58]. In addition, other RLCK VII members, like OsRLCK118 and OsRLCK176, participate in ROS generation by phosphorylating the NADPH oxidase OsRbohB [59].

Although successful pathogens can escape the first layer of plant innate immunity by transferring effector proteins to host plants, plant cells employ NLRs to recognize these effectors both directly and indirectly, and initiate ETI to drive the infected cell to programmed cell death. For example, AvrPphB secreted by *Pseudomonas syringae* cleaves RLCK PBS1 to suppress PTI [24,60]. However, this process could be monitored by NLR protein resistance to pseudomonas syringae 5 (RPS5) and elicits RPS5-specified ETI [60]. Likewise, the effector protein AvrRpt2 activates NLR protein resistance to pseudomonas syringae 2 (RPS2) through eliminating the RPS2-bound RIN4 protein in *Arabidopsis* [61,62]. Additionally, the tomato NLR protein Prf associates with Pto kinase to recognize the effector protein AvrPto [63]. Recently, a pan-genomic analysis of numerous *Pseudomonas syringae* strains identified many unique effector protein sequences that are surprisingly recognized by only a small number of NLRs, suggesting a pervasive role for ETI [64]. Notably, the molecular mechanisms underlying plant NLR activation have recently begun to be revealed by a series of elegant studies that reconstituted and solved the structure of *Arabidopsis* NLR receptor HOPZ-ACTIVATED RESISTANCE 1 (ZAR1). In two recent seminal articles [9,12], the authors first investigated the 3D structure of the ZAR1-RKS1 complex and showed that the ZAR1-RKS1-PBL2^UMP^ complex induced the replacement of an ADP molecule by an ATP molecule with ZAR1, resulting in the full activation of this complex and formation of the pentameric ZAR1 resistosome [9,65]. More importantly, the ZAR1 resistosome has been shown to form pentameric complexes in the plant cell plasma membrane, where it acts as a calcium-permeable channel triggering plant immune signaling [11]. The discovery of the ZAR1 resistosome is a breakthrough in our understanding of NLR-mediated immunity.

In summary, it is widely accepted that the RLP/RLK-RLCK signaling module plays important roles in MAMP-triggered immune responses. However, the exact contributions of most RLCK members to immune signaling still require further clarification. In addition, the immune signaling pathways are also controlled by negative regulators. For example, the complex formed from RLCK PBL13 and an E3 ligase PIRE phosphorylates RbohD and enhances its ubiquitination to negatively regulate ROS production during immunity [66]. Moreover, great efforts are also needed to uncover how NLR proteins recognize various effectors and initiate immune responses.

## 3. Interplay between MAMP- and Effector-Triggered Immunity

Remarkably, several recent studies have suggested that PRR- and NLR-mediated signaling extensively cross-talk to significantly influence immune response outcomes [10,67,68,69,70,71,72]. For example, the NLR protein recognition of Peronospora parasitica 2 (RPP2)- and RPP4-mediated ETI-associated pathogen restriction in *Arabidopsis* against *Hyaloperonospora arabidopsidis* (*Hpa*) races *Emoy2* and *Cala2* requires the RLK/PLP co-receptors BAK1 and BKK1 [37]. Furthermore, avrRpt2 activates RPS2-dependent ETI in wild-type plants [61,62] but fails to elicit effective ETI in *fls2*/*efr*/*cerk1* and *bak1*/*bkk1*/*cerk1* triple mutants in *Arabidopsis* [68,73]. Moreover, BIK1 has recently been identified to be required for ROS burst, immune gene expression, and resistance during ETI [68]. Consistently, pre-activation of ETI by AvrPps4 increases the flg22-induced production of ROS, suggesting that ETI enhances PTI responses [74]. In addition, it has been recently shown that MPK3/6 control the SA component of defense through the expression of some NLR genes, thereby bridging PTI and ETI [69]. Similarly, activation of TIR signaling fosters PTI, as demonstrated by a recent study showing that PTI is attenuated in plants with reduced NLR protein levels [70]. Taken together, there is increasing evidence that PTI and ETI cooperate to guarantee robust immunity, although the precise molecular mechanisms still need further exploration.

## 4. Establishment of Symbiosis

The symbiotic interaction between rhizobia and leguminous plants involves multiple processes that begin with pre-infection events in the rhizosphere [75]. Under nitrogen-limiting conditions, flavonoids are produced by legume roots and are secreted into the rhizosphere to recruit symbiotic partners [76]. When flavonoids are detected by rhizobia, lipochitooligosaccharides (LCOs), known as Nod factors, are synthesized [75,77,78]. In symbiosis, Nod factors are also perceived by LysM-RLKs. In *Lotus japonicus*, Nod factors are recognized by NFR1 kinase and NFR5 pseudokinase, whereas the kinase and pseudokinase are LYK3 and NFP, respectively, in *Medicago truncatula* [79,80,81,82]. The perception of Nod-LCOs elicits downstream signal-transduction [83], which induces the formation of infection threads and nodule organogenesis [84]. Another important signaling pathway involved in legume-rhizobium symbiosis is the type III secretion system (T3SS). The T3SS is widely found in pathogenic bacteria [85,86], and it delivers virulence effectors to host plants to manipulate host biological processes [87]. Interestingly, some rhizobia possess T3SSs, which can be induced by flavonoids to secrete nodulation outer proteins [88], thereby suppressing plant defense reactions. Nonetheless, some nodulation outer proteins activate plant immune responses [88,89,90]. For example, nodulation outer protein L (NopL) secreted by *Rhizobium* sp. NGR234 decreases the protein level of chitinase and therefore suppresses the transcription of resistance genes in both *Nicotiana tabacum* and *Lotus japonicus* [88]. In addition, nodulation outer protein M (NopM) is also secreted by *Rhizobium* sp. NGR234 and it has E3 ubiquitin ligase activity in vitro. Transgenic *Nicotiana benthamiana* plants that express *nopM* exhibit suppressed flg22-induced PTI. However, NopM induces the expression of resistance genes in the transgenic plants [89]. All these data suggest that signaling pathways involved in immune responses are tightly regulated in the establishment of symbiosis.

Similarly, the establishment of AM symbiosis requires presymbiotic communication which depends on mutual signal exchange between fungi and host plants. The recipient plant root extrudes strigolactones (SLs), a class of phytohormone, which act as stimulants of hyphal branching and fungal metabolism [91]. Simultaneously, AMFs secrete Myc factors including sulfated and nonsulfated mycorrhizal-lipochitooligosaccharides (Myc-LCOs) and short-chain COs, such as CO4 and CO5, to elicit presymbiosis responses in the host [92,93]. Both Myc-LCOs and COs are chemically closely related to the MAMPs CO7 and CO8, which elicit plant PTI [43,94,95,96]. It has been shown that OsCERK1 is involved in AM symbiosis [97,98] and recognition of COs, as shown in a study in which CO-dependent nuclear Ca^2+^ spiking was impaired when OsCERK was mutated in rice [99]. Furthermore, OsCERK1 homologs in *Medicago truncatula* and *Pisum sativum*, MtLYK9 and PsLYK9, also function as bifunctional receptors that participate in AM symbiosis and defense responses [100,101].

Importantly, given that OsCERK1 is unable to bind to CO4 and CO5, it is therefore likely that there are additional proteins other than CEBiP and NFR5 that associate with OsCERK1 for AMF ligand perception [102]. Recently, Myc factor receptor 1 (OsMYR1) was identified as an OsCERK1 binding partner for detecting Myc-CO4, as demonstrated by the finding that the Osmyr1 mutant had low colonization with the AM fungus and decreased symbiotic responses [103]. The same group also determined the molecular mechanism by which the OsMYR1/OsCERK1 complex elicits symbiosis rather than an immune response [104]. Briefly, the symbiotic receptor OsMYR1 was shown to disrupt the formation of the OsCERK1-OsCEBiP complex and stop OsCERK1 from phosphorylating the downstream substrate OsGEF1, thereby decreasing the sensitivity of rice to MAMPs [104]. The perception of Myc factors by RLKs is thought to contribute to the establishment of AM symbiosis through activating symbiotic responses including robust nuclear Ca^2+^ oscillations in the rhizodermis and transcriptional regulation of AM symbiosis-associated genes [105,106].

The structural similarity of LysM-RLKs or the dual functions of the same RLK have prompted the question of how RLKs initiate specific signaling for symbiosis or immunity. Recently, several studies have begun to answer this question. An elegant study performed in *Lotus japonicus* and *Medicago truncatula* revealed that the ectodomain of chitin-binding receptor LjLYS6/MtLYK9 enables the kinase to bind long COs such as CO6, CO7, and CO8 instead of CO4, indicating the importance of the ectodomain in discriminating immunogenic and symbiotic factors [107]. Subsequently, another elegant study identified that two diverging motifs in the LysM1 domain of NFR1 and CERK6 are responsible for discriminating between immunity and symbiotic functions in *Lotus japonicus* [41]. The molecular mechanisms governing specific recognition of distinct signals by LysM receptors across different plant species still need further investigation. In addition, a lysin motif effector, RiSLM, has been shown to protect chitin from chitinases, and in doing so it efficiently interferes with chitin-triggered immunity to facilitate arbuscular mycorrhizal symbiosis [108]. In summary, the strategies used by plants to distinguish pathogenic from symbiotic microbes are complicated and still need investigation. The comparative analysis of different model plants using proteomics, transcriptomics, and structural analysis would help deepen our understanding of immunity and symbiosis.

## 5. Tight Regulation of Plant Immunity during Symbiosis

Several studies have now shown that the establishment of symbiotic associations is always accompanied by a transient immune response [109,110,111]. For example, a transcriptome analysis of *Bradyrhizobium japonicum*-inoculated soybean showed strong induction of plant defense-related genes in the 12 h after inoculation but with expression returning to the baseline within 24 h [109]. Similarly, AM fungus *Glomus intraradices* colonization increased the transcriptional level of defense genes in rice leaves [111]. Therefore, the symbiotic associations appear to be established on the basis of tightly regulated immunity. Here, we focus on how plant immunity is tightly controlled during symbiosis.

**Figure 2:**
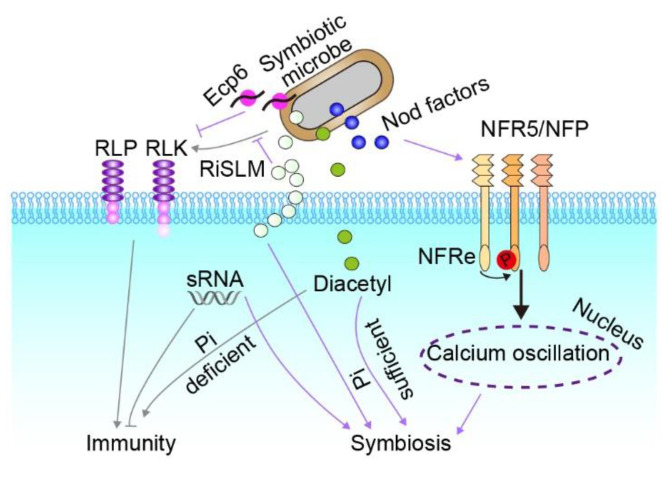
Signaling pathways employed by microbes to evade plant immunity and establish symbiosis. Beneficial microbes establish symbiotic interactions by secreting effectors that interfere with plant immune systems. Nod factors, detected by NFR5/NFP, trigger calcium oscillations in the nucleus and promote symbiosis. Recently, a new LysM receptor, NFRe, has been identified and guarantees robust symbiotic signaling through phosphorylating NFR5. Furthermore, RiSLM, a lysin motif effector, suppresses recognition of microbes by plants by protecting the cell walls from chitinases. Similarly, the Ecp6 protein, which contains the LysM domain, competes with plant chitin receptors for ligand binding to prevent host immune activation. Moreover, sRNAs from microbes have been found to impact the host immune system and help establish symbiosis. In addition, microbial diacetyl modulates the transition from immunity to symbiosis in a phosphate-dependent manner.

Symbionts can evade recognition by the plant through MAMP divergence or effectors that actively suppress defense responses (Figure 2). Bacterial polysaccharides, as polyanionic molecules, can chelate calcium ions and thus suppresses innate immunity to enhance symbiosis [112,113]. In line with this, roots inoculated with an exopolysaccharide-deficient mutant of *Sinorhizobium meliloti* express upregulated plant defense genes compared to roots inoculated with the wild type [113]. Furthermore, NFs have been shown to partially inhibit PTI in a nodulation-independent manner, and this inhibition occurrs in both legumes and non-legumes [114]. In addition, some LysM receptors, such as the newly identified epidermal LysM receptor (NFRe), guarantee robust symbiotic signaling through phosphorylating NFR5, which in turn regulates NFRe downstream signaling. Consistently, mutants of *Nfre* show an increased calcium spiking interval and fewer nodules in the presentence of rhizobia [84]. Moreover, diacetyl, a bacterial volatile compound, can transit immunity to symbiosis depending on phosphate availability. Diacetyl partially inhibits the ROS burst and fosters symbiont colonization while phosphate is abundant. However, diacetyl could enhance hormone-mediated immunity under phosphate-deficient conditions [115]. Another important strategy by which microbes invade the host plant immune system is by interfering with plant PRR function via the delivery of effector proteins or small RNAs to the apoplast or the host cell [116,117,118]. One example is Ecp6, a protein containing the LysM domain, which competes with plant chitin receptors for ligand binding to prevent host immune activation through PRR ligand deprivation [119,120,121]. In addition, a recent study revealed that the symbiosis receptor-like kinase (SymRK) associates with LjBAK1 to directly inhibit LjBAK1 kinase activity and thus suppress innate immunity [122]. Co-infection of the legume *Medicago truncatula* with rhizobium and pathogenic bacteria shows that nodules display a weak defense response upon pathogen infection and provide a diffusion barrier to prevent pathogens from spreading to the rest of the plant [123], indicating spatiotemporal suppression of PTI during symbiosis. It is of great significance to investigate how plants maintain immune responses in a spatiotemporally specific manner while employing distinct strategies for plant–microbe symbioses.

Plants also seem to directly suppress immunity signaling with endogenous small RNAs (sRNAs) to promote symbiotic associations [124]. For example, upon rhizobia infection, miR169 binds to polysomes and is decreased in polysomal complexes to accelerate nodulation [125]. In another example, miR171b, expressed in arbuscule-containing cells, promotes symbiosis as well [126]. Briefly, LOST MERISTEMS 1 (LOM1) is identified to be a positive regulator of AM symbiosis and is a substrate of miR171 family members. However, miR171b has a mismatched cleavage site and lacks the ability to downregulate the transcription level of *LOM1*. Moreover, miR171b expression protects the *LOM1* gene from silencing by other miR171 family members. Moreover, high-throughput sRNA sequencing of maize roots identified many miRNAs that regulate symbiosis via lipid metabolism, phosphate starvation, or fatty acid metabolism [127]. For example, decreased levels of miR399 are able to inhibit the expression of Pi starvation-inducible genes in the direct Pi-uptake pathway, leading to reorganization of Pi uptake. Additionally, the decreased expression of several miRNAs, such as zma-miR399b-5p, results in an increase of fatty acid biosynthetic process genes’ expression, which promotes fatty acid metabolism and lipid delivery from plants to AM fungi. A recent study reported that transfer-RNA-derived sRNA fragments from rhizobial bacteria modulate host nodulation-associated genes by employing the host’s RNAi machinery [128]. This transfer of sRNAs and subsequent gene silencing in the target organism is known as cross-kingdom RNAi. Although many miRNAs have been reported to function in symbiosis, whether symbiotic microbes transfer sRNAs to the host plant to repress plant immunity is largely unknown.

## 6. Conclusion and Perspectives

Recent studies have provided a detailed understanding of plant immunity. Microbial MAMPs and/or effectors sensed by host plants trigger PTI and/or ETI. Studies of the PRR and NLR networks have revealed that the perception of pathogenic invasion is highly complex and probably requires the cooperation of multiple immunogenic signals [12]. Similarly, accumulating evidence indicates that a successful response requires complex cooperation of multiple pathways

The establishment of mutualistic symbiosis requires beneficial microbes to either avoid producing MAMPs, repress plant immunity, or both. Although some effector proteins or small RNAs have been found to negate plant immunity, there are also more challenging questions that relate to the interplay between immunity and symbiosis signaling. For example, how do cross-kingdom sRNAs work synergistically? How does the plant cell respond to many diverse signals received simultaneously? Are there any other strategies employed by microbes to restrain host plant immunity? To solve these questions, transcriptomic and proteomic analyses might be useful to screen for differentially expressed genes and proteins at the global level during different stages of infection. Moreover, single-cell sequencing technology is likely to be useful for identifying novel genes and proteins involved in immunity and symbiosis at the single-cell level. Furthermore, a novel technology called proteolysis-targeting chimeras (PROTACs), which degrade targeted proteins, could help us investigate the functions of newly identified proteins [129,130]. In addition, we cannot ignore the roles of protein post-translational modifications, such as ubiquitination, phosphorylation, and acetylation, in plant–microbe interactions.

There has historically been a focus on the early stages of symbiosis and plant immunity, but it is also interesting and necessary to understand the maintenance and termination of these processes. Plants respond to invading microbes through complicated networks, but clearly and comprehensively understanding these plant–microbe interactions will provide the foundation to engineer disease resistance genes into important crops and help increase crop yield.
